# Design, Development, and Optimization of Dexibuprofen Microemulsion Based Transdermal Reservoir Patches for Controlled Drug Delivery

**DOI:** 10.1155/2017/4654958

**Published:** 2017-09-27

**Authors:** Fatima Ramzan Ali, Muhammad Harris Shoaib, Rabia Ismail Yousuf, Syed Abid Ali, Muhammad Suleman Imtiaz, Lubna Bashir, Shazia Naz

**Affiliations:** ^1^Department of Pharmaceutics, Faculty of Pharmacy, University of Karachi, Karachi, Pakistan; ^2^H.E.J. Research Institute of Chemistry, International Center for Chemical and Biological Sciences, University of Karachi, Karachi, Pakistan

## Abstract

The aim of the study was to develop a reservoir-type transdermal patch for a controlled delivery of dexibuprofen and to evaluate its in vivo anti-inflammatory activity in Albino Wistar rats. In order to develop these patches, six formulations of dexibuprofen microemulsion comprising ethyl oleate, Tween 80: PG (2 : 1), and water were prepared by simplex lattice design and characterized. The reservoir compartment was filled with these microemulsions and in vitro release and skin permeation were assessed. The optimized patch was obtained on the basis of the responses: *Q*_24_ and flux. The impact of drug loading, surface area, membrane thickness, adhesive, and agitation speed on drug release and permeation was also studied. The skin sensitivity reaction and in vivo anti-inflammatory activity of optimized patch were evaluated. Stability study at three different temperatures for three months was carried out. The result suggests that a membrane based patch with zero-order release rate, *Q*_24_ of 79.13 ± 3.08%, and maximum flux of 331.17 *µ*g/cm^2^h can be obtained exhibiting suitable anti-inflammatory activity with no visible skin sensitivity reaction. The outcomes of stability study recommend storage of patches at 4°C having shelf-life of 6.14 months. The study demonstrates that the reservoir-type transdermal patch of dexibuprofen microemulsion has a potential of delivering drug across skin in controlled manner with required anti-inflammatory activity.

## 1. Introduction

Transdermal systems deliver drugs across skin into systemic circulation and are considered as one of the suitable routes for drug administration. These can be used for numerous clinical indications [1]. Human skin provides an effective barrier against chemical penetration of drugs, and minimizing this hindrance is the target of most of the transdermal preparations [2].

Microemulsions are effective drug delivery vehicles for topical and transdermal preparations [3]. These preparations increase cutaneous delivery of drug by increasing solubility of both hydrophilic and lipophilic molecules; as a result concentration gradient is increased towards skin. The components of microemulsion also have permeation enhancing property [4]. Therefore, microemulsions can be effectively used to enhance the permeation of drug across skin.

Reservoir-type transdermal patches enclose drug in a rate-controlling membrane and deliver drug by zero-order rate process and possess certain advantages over other types of patches; such that they have design flexibility and effective control on release rates [5]. In the current study, a reservoir-type transdermal patch consisting of dexibuprofen microemulsion was formulated for effective and controlled delivery of drug through skin. Ibuprofen, an arylpropionic acid NSAID, possesses antipyretic, analgesic, and anti-inflammatory activities and is considered as over-the-counter drug. S(+)-isomer also called dexibuprofen is more potent as compared to racemic ibuprofen [6]. Ulceratic perforation and gastrointestinal bleeding are common adverse effects of NSAIDs. Dyspepsia is also commonly observed side-effect of ibuprofen and NSAIDs. The symptoms of dyspepsia include heartburn, abdominal pain, anorexia, and distention [7].

Recent efforts are focused on formulating a reservoir-type transdermal patch filled with microemulsion of dexibuprofen. Dexibuprofen having log *P* value of 3.97 [8] and biological half-life of 1.8–3.5 h [9] possesses suitable physicochemical and pharmacokinetic properties making it potential candidate for transdermal preparation.

The goal of this study is to formulate a membrane based transdermal system of dexibuprofen microemulsion. This delivery system will release the drug in a controlled manner with efficient permeation to achieve required anti-inflammatory activity and precluding adverse effects associated with gastrointestinal tract.

## 2. Materials and Methods

### 2.1. Materials

Dexibuprofen was gifted by Shasun Pharma Industry, India. Other excipients/chemicals/reagents used were ethyl oleate (EO) and Tween 60 (Avonchem, Cheshore, UK), Tween 40, Tween 80, triethanolamine and ethanol (BDH, Poole, England), propylene glycol (PG) and polyvinyl alcohol (PVA) (Daejung, Gyeonggi-do, Korea), polyethylene glycol 400 (PEG 400) (Sigma Aldrich, Steinheim, Germany), and methanol (TEDIA, Fairfield, USA). The patch components backing membrane (3M-9720), rate-controlling membrane (3M-CoTran 9728 (2 mil) and 9716 (4 mil)), and release liner (SCOTCHPAK 9755) were gifted by 3M, St. Paul, USA. Acrylate adhesive Duro-Tak 387/2510 was supplied by Henkel Corporation (Bridgewater, USA).

### 2.2. Animals

The animals used for in vitro skin permeation, skin sensitivity, and in vivo anti-inflammatory studies were Albino Wistar rats (150–180 g) obtained from the animal house, Faculty of Pharmacy, University of Karachi, Pakistan. All ARRIVE guidelines for the care and use of laboratory animals were followed. The animals were kept in a controlled environment (25 ± 1°C) and free access to food and water was provided.

### 2.3. Screening of Microemulsion Components

To find out the suitable components for dexibuprofen microemulsion, the solubility of dexibuprofen in oil (ethyl oleate), surfactants (Tween 40, 60, and 80 and triethanolamine), and cosurfactant (PG, PVA, PEG 400, and ethanol) was determined through method reported by Roni and Jalil [10]. An excess of drug was taken in 5 g oil/surfactant/cosurfactant, which was then shaken for 15 minutes in a vortex mixture (Whirl Mixer Lab, England) and stored overnight at room temperature. After 24 hours the sample was centrifuged (Heraeus Labofuge 200, Osterode, Germany) at 3000 rpm for 5 minutes. Supernatant was collected and diluted with methanol. The diluted sample was filtered using Whatman 102 and further diluted with methanol. The concentration of saturated solution was determined through UV-spectrophotometer (UV-1800, Shimadzu Corporation Kyoto, Japan) at 225 nm using methanol as a blank.

The solubility was also determined in oily mixture of ethyl oleate, Tween 80: PG in a ratio of 1 : 10 : 5 according to the method reported by Chen et al. [11]. Dexibuprofen was taken in excess in the mixture and stirred magnetically for 72 h at 25°C. Sample was collected and centrifuged for 10 minutes at 5000 rpm. The supernatant was diluted and filtered. The sample was further diluted and analyzed by UV-spectrophotometer at 225 nm. All experiments were performed in triplicate.

### 2.4. Construction of Pseudoternary Phase Diagram

Water titration method was used to determine the range of concentration of components at which microemulsion can be formulated [11]. The weight ratios of 1 : 1, 2 : 1, and 3 : 1 of Tween 80 and propylene glycol were used for the construction of phase diagram. For each weigh ratio, the oil to mixture of surfactant and cosurfactant ratio was kept, 0.5 : 9.5, 1 : 9, 1.5 : 8.5, 2 : 8, 2.5 : 7.5, 3 : 7, 3.5 : 6.5, 4 : 6, 4.5 : 5.5, 5 : 5, 5.5 : 4.5, 6 : 4, 6.5 : 3.5, 7 : 3, 7.5 : 2.5, 8 : 2, 8.5 : 1.5, 9 : 1, and 9.5 : 0.5. The oil and mixture of surfactant and cosurfactant were weighed accordingly and vortexed and then water was added dropwise under constant stirring. After equilibration, the samples were visually inspected and categorized as microemulsion, emulsion, or gel. The phase diagram was constructed using Chemix School 3.5.1.

### 2.5. Formulation of Dexibuprofen Microemulsion

Simplex lattice design experiment was used to determine the composition of microemulsion formulation as shown in Table 2. This method was reported by many scientists for formulation of three component systems [12–16]. Surfactant mixture of Tween 80 and propylene glycol (2 : 1) was prepared. Microemulsion system was formulated by mixing drug (10%), oil (*X*_1_) and surfactant mixture (*X*_2_) together. Water (*X*_3_) was precisely added dropwise to these oily mixtures with gentle magnetic stirring. After the system was equilibrated magnetic stirring was continued for 30 minutes.

### 2.6. Characterization of Microemulsion

Physical characterization of microemulsion was done on the basis of pH, conductance, viscosity, and refractive index. The pH of the formulated microemulsion was determined using pH meter (Mettler MP-220, Schwerzenbach, Switzerland) by dipping the glass electrode in the emulsion to be tested. The conductivity meter (WPA-CMD-500, Cambridge, UK) was used to measure the electromotive conductivity. Brookefield-type viscometer (Haake-19, Karlsruhe, Spain) was used to determine the viscosity; L1 spindle was set at 60 rpm. The refractive index was determined through Abbe's refractometer (Schmidt Haensch-24298, Germany) by placing a drop on glass slide and scale was read. All the measurements were made at 25°C in triplicate. The results were measured as mean ± standard deviation [17].

### 2.7. Droplet Size Analysis

Droplet size of microemulsion was determined through Dynamic Light Scattering (DLS). DLS measurements were performed as recently described by Hameed et al. [18]. Briefly, laser spectroscatter-201 system with a He-Ne laser providing a 690 nm light source and an output power in the range of 10–50 mW was used.

For all drop size measurements, an autopiloted run of 50 measurements at every 20 s, with a wait time of 1 s, was conducted at 25°C. Samples (20 *µ*L) were directly introduced into a special quartz SUPRASIL® cell (light path 1.5 mm, Hêllma, Germany) for measurements. The scattered light was collected at a fix scattering angle of 90°. The autocorrelation functions were analyzed using the CONTIN program to obtain hydrodynamic radius (*R*_*H*_) distributions. *R*_*H*_ is related to the diffusion coefficient by the Einstein–Stokes equation. The data were analyzed using XtalConcepts software (XtalConcepts, Germany) provided with the instrument. The impact of time on droplet size of optimized formulation was also studied for 6 hours.

### 2.8. Drug Content Evaluation

The content of dexibuprofen in microemulsion was determined through UV-spectrophotometer by modifying the assay method reported by Kim et al. [19]. The weighed quantity of microemulsion was dissolved completely in methanol. The solution was sonicated for 10 minutes. Volume was made up to obtain the concentration of 100 *µ*g/mL. This solution was further diluted and analyzed at 225 nm spectrophotometrically and percent drug content was determined by comparing the absorbance with that of standard.

### 2.9. Stability of Microemulsion

The stability of microemulsion was determined for 6 months by clarity and phase separation analysis. The samples were kept at 32 ± 0.2°C. The content of dexibuprofen in microemulsion was determined monthly. Physical stability was tested by centrifugation test, in which the microemulsions were centrifuged at 5000 rpm for 1 h.

### 2.10. Dose Calculation for Transdermal Administration

The delivery of active agent from controlled release devices achieves constant levels approximately. This requires comparatively less drug to produce required action in the mentioned duration of time than conventional dosage form. Baker describes the equation for the calculation of dose (*M*_*o*_) delivered for a required duration of action (*t*_*e*_) from controlled release system considering *M*_*e*_ as minimum effective concentration and *t*_1/2_ is half-life of drug [20].

(1) $$\begin{array}{c} t_{e}=t_{1/2}\frac{M_{o}-M_{e}}{\ln2M_{e}}. \end{array}$$

The minimum effective concentration of dexibuprofen is 11 mg/L; half-life and volume of distribution are 3.5 h and 8.61 L, respectively [21]. The patch was designed to deliver drug for 24 h. The transdermal dose calculated by using (1) was

(2) M0=24×ln⁡2×113.5+11=63 mg/LM0×Vd=63×8.61=500 mg.

### 2.11. Fabrication of Reservoir Patch of Dexibuprofen

The reservoir-type transdermal patch of dexibuprofen microemulsion was formulated by heat sealing technique. The backing layer and rate-controlling EVA membrane were heat sealed at 90°C. The microemulsion was filled in the device through disposable syringe. The unsealed side was heat sealed again. The patch was then cut to appropriate size. It was ensured that the device does not show any leakage. The patch was stored in the aluminium foil pouches at room temperature.

### 2.12. Content Uniformity of Patches

The reservoir compartment containing drug was opened and extracted with 100 mL of methanol through 30 minutes of sonication. The resultant solution was filtered, and 0.5 mL of this sample was diluted with 100 mL methanol. The drug content of 10 patches of each formulation was measured using UV-spectrophotometer at 225 nm. The content of each formulation was determined and acceptance value was calculated as reported in USP 34 [22].

### 2.13. In Vitro Release Study

In vitro release of dexibuprofen from reservoir patch was determined by using USP apparatus 5 (paddle over disk) (Erweka DT-600, Heusenstamm, Germany). The 500 mL of phosphate buffer pH 7.4 was placed in the vessel. Temperature was adjusted to 32 ± 0.2°C. The patch was placed on USP transdermal sandwich (90 mm diameter, 17′′ mesh) (Labecx, Santa Clarita, California, USA) and immersed into medium; such that the patch was placed flat and rate-controlling membrane faced upward. The rotation of paddle was adjusted at 100 rpm. Aliquots of 10 mL were withdrawn and replaced with medium at specified time points, that is, 30 min, 1, 2, 4, 6, 8, 10, 12, and 24 h. The samples were diluted and analyzed through spectrophotometer at 225 nm. The experiment was performed in triplicate. Mathematical models such as zero order, first order, Higuchi, Korsmeyer-Peppas, Weibull, and Makoid Banakar (see (3)–(8)) were applied to determine the release kinetics. The coefficient of correlation and rate constants were determined using Add-In Program DD Solver®. The effect of variable drug loading, surface area, membrane thickness, adhesive, and agitation speed on release from optimized patch was also studied.

(3) $$\begin{array}{c} Q_{t}=Q_{o}+k_{0}t, \end{array}$$

where *Q*_*o*_ represents the initial amount of drug in the dosage form, *Q*_*t*_ is the amount of drug released at time *t*, and *K*_*o*_ is a zero-order rate constant.

(4) $$\begin{array}{c} \log Q_{t}=\log Q_{o}+\frac{k_{1}t}{2.303}, \end{array}$$

where the drug released at particular time *t* is represented by *Q*_*t*_ and *Q*_*o*_ is the initial amount of drug present in dosage form. *k*_1_ is the first-order rate constant.

(5) $$\begin{array}{c} Q_{t}=K_{H}t^{1/2}. \end{array}$$

Higuchi dissolution constant is represented by *K*_*H*_.

(6) $$\begin{array}{c} Q_{t}=at^{n}, \end{array}$$

where a is a structural and geometric dosage form characteristic, release exponent is expressed by *n*, and it indicates the release mechanism of drug.

(7) $$\begin{array}{c} m=1-\exp\left[\;\frac{-{\left(t-T_{l}\right)}^{b}}{a}\;\right], \end{array}$$

where *m* is drug accumulated fraction in solution at any time (*t*). The scale parameter is, *a*, defining time scale of process. Lag time is presented by *T*_*l*_, that is, the time required before the onset of drug release; in most cases it will be zero. *b* is considered as shape parameter and expresses curve.

(8) $$\begin{array}{c} \frac{M_{t}}{M_{\infty }}=k_{MB}t^{n}e^{\left(-ct\right)}, \end{array}$$

where *K*_MB_, *n*, and *c* are empirical parameter and *M*_*t*_/*M*_*∞*_ is the accumulation fraction of the drug in solution at time *t* [23].

### 2.14. In Vitro Skin Permeation Study

Prodduturi et al. reported the use of USP apparatus 5 for in vitro permeation study of reservoir patches having larger surface area. Since the commonly used equipment for permeation studies is Franz diffusion cell, other types of large patches can be cut to fit the donor chamber of this cell. However, in reservoir-type patch, the patch cannot be cut without losing its integrity [24].

The vessel was filled with 600 mL of Hank's balanced salt solution. The temperature was maintained to 32 ± 0.2°C. The transdermal patch was placed on USP disk such that it was flat and rate-controlling membrane faced upward. The freshly excised full-thickness rat skin was then placed on this patch in a way that epidermal region was placed on patch and dermal region faced upward. Skin integrity was visually inspected prior to the placement. The apparatus was rotated at 100 rpm. Aliquots of 10 mL were withdrawn and replaced with medium at selected time points, that is, 30 min, 1, 2, 4, 6, 8, 10, 12, and 24 h. Each sample was diluted and analyzed spectrophotometrically at 225 nm. Each experiment was performed in triplicate. Flux (*J*) was determined from slope of the linear portion of cumulative amount permeated per unit area (*Q*) versus time (*t*). Lag time (*L*) was determined by extrapolation of line to abscissa. Equation (9) were used to determine permeability coefficient (*P*) and diffusion coefficient (DC) derived from Fick's law of diffusion.

(9) P=JCDC=h26L,

where *C* is the concentration of the drug in patch and h represents the thickness of the skin [25].

The rate of drug delivery is controlled by either device or stratum corneum. The flux observed can be combined effect of both device and skin. Equation (10) was used to determine the fraction rate controlled by device (*F*_*D*_) and skin (*F*_*S*_), respectively. These are computed by comparison of quantity of drug released over a given period of time from device (*M*_device_) to when it is in contact with skin (*M*_total_) [26].

(10) FD=MtotalMdeviceFS=1−FD.

The impact of varying drug loading, surface area, membrane thickness, adhesive, and agitation speed on permeation from optimized patch was also studied.

### 2.15. Optimization of Dexibuprofen Reservoir Patch

The simplex lattice design was used for formation of microemulsion. The ranges of independent variables were computed from pseudoternary phase diagram and were set as follows: 5–15% for oil (*X*_1_), 55–65% for (*X*_2_) Smix, and 20–30% for (*X*_3_) water. On the basis of these concentration ranges, 6 runs were generated using Design-Expert® version 7 (Stat-Ease, Inc., Minneapolis). The formulated reservoir patches were optimized on the basis of corresponding responses, that is, release of drug from patch at 24 h (*Q*_24_) (*Y*_1_) and flux (*Y*_2_). Response surface curve for these responses was constructed.

### 2.16. Skin Sensitivity Test

The skin sensitivity test was performed according to the method reported by Amrish and Kumar [27]. The animals were kept in a cage for 7 days before test to acclimatize with the environment. Dorsal abdominal skin of rat was shaved using electric clipper 24 h before experiment. The optimized patch was placed on skin. The patch was removed 24 h after application. Signs for allergic conditions or irritation were visually examined. Experiments were performed in triplicate.

### 2.17. In Vivo Anti-Inflammatory Study

To evaluate the anti-inflammatory activity of optimized microemulsion reservoir patch, carrageenan hind paw edema test reported by Chandra and Sharma [28] with modification was carried out on Albino Wistar rats (150–180 g). Briefly, the rats were kept in fasting condition and were only provided with water overnight. The animals were divided into two groups (control and test) each group having 6 rats. The control group only received carrageenan injection. The 4 cm^2^ optimized reservoir patch of dexibuprofen (1 mg) was applied on the left hind paw of the test rats. After 2 h, 10 mL of carrageenan solution (1%) was injected in left hind paw of both groups to induce inflammation. The swelling of paw of both control (*S*_*c*_) and test groups (*S*_*t*_) was measured using Vernier calliper (Seikobrand, China) for 4 h after carrageenan injection. The percent edema inhibition was determined by using

(11) $$\begin{array}{c} \%\text{ Inhibition}=\frac{S_{c}-S_{t}}{S_{c}}\times 100. \end{array}$$

### 2.18. Stability Studies

The stability studies were performed according to the method described by Pichayakorn et al. [29]. The optimized reservoir-type patch of dexibuprofen was stored under three different conditions, that is, 4°C, room temperature, and 45°C for 3 months. For analyzing dexibuprofen content in patch the reservoir compartment was opened and extracted with methanol as specified in Section 2.12. The concentration was determined spectrophotometrically at 225 nm. Drug release and permeation were also determined. The stored samples were analyzed monthly in triplicate.

## 3. Result and Discussion

### 3.1. Solubility in Microemulsion Components

In a previous study conducted by Chen et al., ethyl oleate was used as an oil phase for microemulsion of ibuprofen having solubility of 0.153 ± 0.0009 g/mL [11]. The solubility of dexibuprofen in ethyl oleate was found to be 0.182 ± 0.011 g/mL. Therefore, it was selected as an oil phase. The surfactant and cosurfactant were also selected on the basis of solubility of drug in these vehicles. Zhao et al. also performed the solubility studies for obtaining the suitable surfactant and cosurfactant for ropivacaine microemulsion [30]. As shown in Table 1, among the four surfactant and cosurfactant studies Tween 80 and PG have highest solubility of 0.306 ± 0.006 g/mL and 0.209 ± 0.026 g/mL, respectively. Therefore, these were fixed for further studies.

The solubility of 0.536 ± 0.013 g/mL was found in the oily mixture comprising ethyl oleate, Tween 80, and PG which is greater than the solubility of ibuprofen in similar oily mixture, that is, 0.439 ± 0.017 g/mL [11].

### 3.2. Phase Diagram

**Figure 1:**
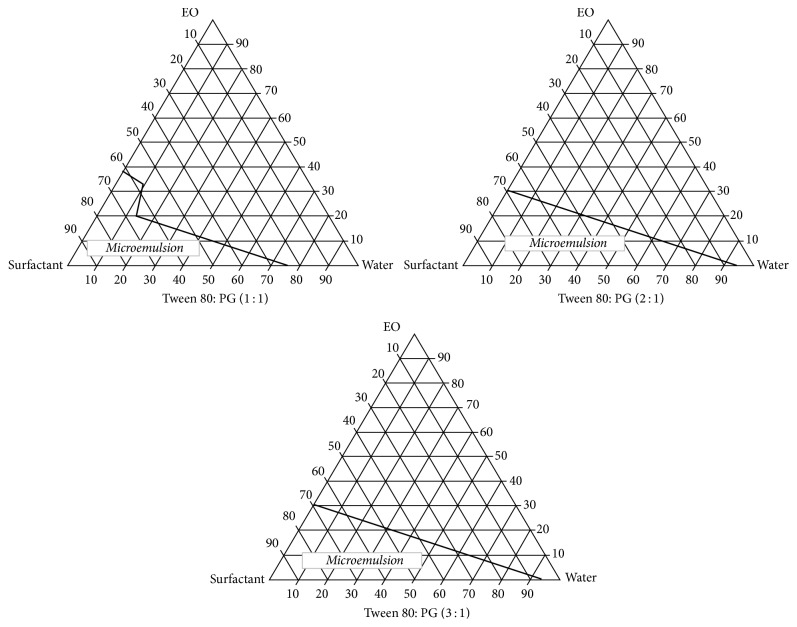
Pseudoternary phase diagram of oil (ethyl oleate), surfactant mixture (Tween 80: PG), and water at 25°C in weight ratio of 1 : 1, 2 : 1, and 3 : 1.

Mixing of components in exact ratio is essential for formulating microemulsion. Variation in concentration of components leads to phase separation [31]. Construction of pseudoternary phase diagram is therefore necessary to determine the exact composition of components for microemulsion preparation [32]. Phase diagram as represented in Figure 1 was constructed for variable weight ratio of Tween 80: PG, that is, 1 : 1, 2 : 1, and 3 : 1. The microemulsion region is presented in the diagram and rest of the region represents formation of emulsion or gel. The microemulsion region computed was comparable to the findings of Chen et al. [11]. In the weight ratio of 2 : 1 wider concentration range was forming microemulsion; thus, it was selected for development of microemulsion.

### 3.3. Characterization of Microemulsion

Table 2 represents the physical characteristics of microemulsion. pH was found to be between 4.66 ± 0.04 and 5.46 ± 0.02. An ideal pH for skin preparations must be between 5 and 6, as the acidic pH leads to skin irritation while the basic pH promotes microbial growth on skin [33]. The pH of formulations F4, F5, and F6 was found within this range.

On the basis of conductance, the type of microemulsion, that is, w/o or o/w, can be determined [34]. The conductance of less than 100 *µ*S/cm specifies preparation of water in oil microemulsion [35]. The conductance of formulation ranged between 12.71 ± 0.02 *µ*S/cm and 32.7 ± 0.81 *µ*S/cm indicating w/o microemulsion. The refractive index of microemulsion was found to be 1.44 and 1.45 which is similar to the refractive index of ethyl oleate thus, also indicating the water in oil microemulsion. As the quantity of oil increases in the formulation, refractive index also increases from 1.44 to 1.45. This is similar to the finding of Moghimipour et al. where the refractive index values indicate formation of w/o microemulsion [36].

Microemulsions exhibit Newtonian flow property [37]. This was also found in the study that viscosity remained constant at variable rate of shear, that is, exhibiting Newtonian behaviour. The viscosity values of formulation F1–F6 ranged between 110 ± 15.27 mPa·S and 360 ± 15.27 mPa·S.

The percentage of drug content of microemulsion formulations was found between 97.9% and 100.8%, which is within the specified limit, that is, 90–110%.

### 3.4. Droplet Size of Microemulsion

The droplet size of formulations F1–F6 was found to be between 119.8 ± 13 nm and 221.6 ± 30 nm with PDI between 0.35 and 0.56. In a study conducted by Biswal et al., the droplet size of lornoxicam microemulsion was found to be between 175.7 nm and 305.1 nm with PDI of 0.289 and 0.894 [38]. The particle size of microemulsion depends on several mechanisms. As the surfactant concentration increases, the droplet size decreases. Oil and cosurfactant concentrations also affect the particle size [33]. The microstructure may also be altered due to the interaction between loaded drug and microemulsion components. The variety of components used for construction of microemulsion leads to variety of structure and this makes characterization of structure difficult [39]. The fluctuation in droplet of microemulsion is attributed to the difference in energy scale. The energy needed to compress the fluids inside or outside the drop or change the area per surfactant molecule is often much larger than the energy needed to bend the interface leading to droplet fluctuation [40].

**Figure 2:**
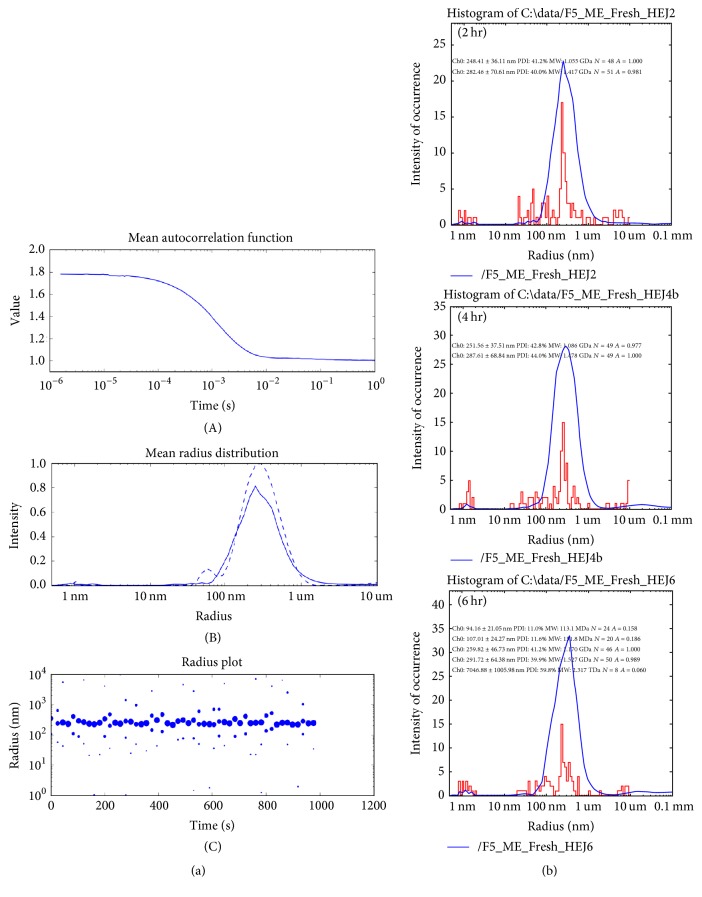
Physical characterization of droplet size of ME by DLS. (a) Dynamic Light Scattering results of freshly prepared F5_ME illustrating the experimental conditions, that is, the mean autocorrelation function, monodispersity, and the radius plots (A) to (C), respectively. (b) Mean corresponding radius of hydration and percent polydispersity followed till 6 hrs clearly revealed increase in radius and polydispersity. All experiments were performed with an autopiloted run of 50 measurements at every 20 s, with a wait time between of 1 s (at 25°C). See Table 3 for details.

Table 3 represents the droplet size and polydispersity index as a function of time indicating the increase in droplet size with time. Cavalli et al. also concluded that the droplet size and polydispersity index of oil-in-water microemulsion increased over time [41].  Figure 2 represents the autocorrelation function, monodispersity, and radius plot of freshly prepared F5 formulation. The 6-hour analysis of mean corresponding *R*_*H*_ is also presented in this figure.

### 3.5. Stability of Microemulsion

The formulated microemulsions kept at 32 ± 0.2°C were stable during the course of 6 months and the percentage drug content of all formulations remained within the mentioned limit. The microemulsions kept for stability studies were clear throughout this period and no phase separation was observed when subjected to centrifugation test. Thus, these preparations exhibited both chemical and physical stability throughout the storage period.

### 3.6. Content Uniformity of Reservoir Patches

United States Pharmacopeia specifies testing uniformity of dose content for transdermal patches, and the maximum acceptance value allowed for Level 1 is 15 [22]. The individual % drug content of 10 samples and acceptance value calculated for each formulation were within limit. The mean of % drug content was 99.47 ± 2.5%, 99.67 ± 3.5%, 99.43 ± 4.4%, 98.63 ± 4.37%, 98.981 ± 2.31%, and 99.02 ± 4.8%, respectively. The acceptance value for all the formulations was below 15, that is, within the range, and hence, all formulations passed content uniformity test.

### 3.7. In Vitro Release Study

**Figure 3:**
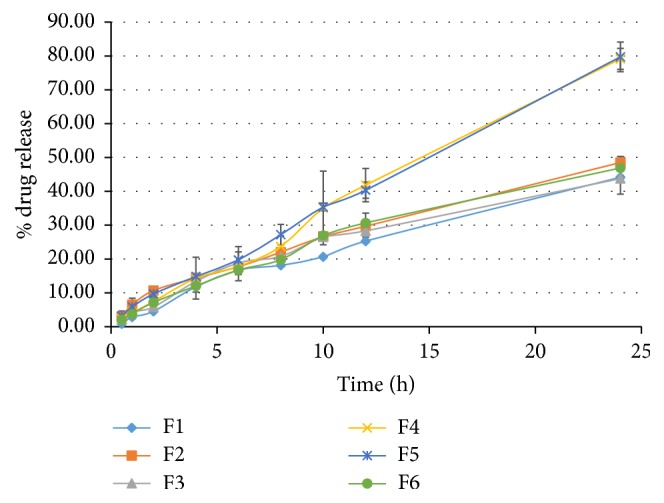
In vitro release profile of formulations F1–F6 from dexibuprofen reservoir patches.

Reservoir patches are diffusion controlled systems and the membrane regulates the drug release from it, which is essential for skin permeation [42]. Drug release as demonstrated in Figure 3 at 24 h is maximum of F4 and F5, that is, 79.13 ± 3.08% and 79.73 ± 4.37%, respectively. Various kinetic models such as zero order, first order, Higuchi, Korsmeyer-Peppas, Weibull, and Makoid Banakar were also applied and F1, F2, F3, and F6 as presented in Table 4 were best complying with Makoid Banakar model with *R*^2^ value of 0.9918, 0.9976, 0.9952, and 0.9962, respectively. This is similar to the estradiol membrane patches, which exhibited Makoid Banakar model [23]. The nicotine reservoir patches comprising natural rubber membrane displayed Higuchi release profile [29]. However, F4 and F5 followed a zero-order release model with *R*^2^ value of 0.9952 and 0.9985, respectively. Reservoir patches display true zero-order release pattern for attaining constant serum drug level [42], which is also observed in case of F4 and F5. *n* of Korsmeyer-Peppas model for all formulations is between 0.5 and 1, thus indicating non-Fickian transport [43]. The value of *k* of Makoid Banakar is approximately zero; in this case the parameter (*n*) becomes similar to Korsmeyer-Peppas release exponent [23]. The value of more than 0.5 also indicates non-Fickian transport.

**Figure 4:**
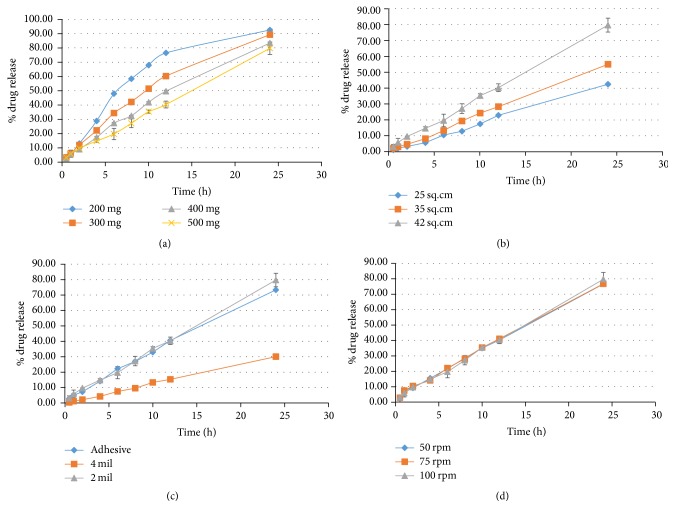
Effect of variable components on release of drug from dexibuprofen reservoir patch. (a) Effect of drug loading on release. (b) Effect of surface area on release. (c) Effect of transdermal components on release. (d) Effect of agitation speed on release.

Drug loading has a significant impact on drug release from transdermal systems. The higher drug loading decreases the rate of diffusion to 50%. Lower drug loading leads to faster drug release [44]. This is in line with the current findings as presented in Figure 4(a) where maximum drug was released from 200 mg patch, that is, 92.45 ± 0.75%. Surface area of patch in contact with skin is another predictor of drug release [26].  Figure 4(b) denotes that the release from higher surface area patch was maximum. This was also concluded by Thacharodi and Rao that decreasing the area also reduces the release of drug from device [45].

The impact of thickness of reservoir-membrane on release was studied and it was observed that the release of drug from 2 mil thick membrane was 79.73 ± 4.37% and 4 mil thick was 30.06 ± 0.3%. This was comparable to the outcomes of Pichayakorn et al. that increasing the membrane thickness reduces the drug release [29]. The adhesive applied on 2 mil thick membrane exhibited release of 73.32 ± 0.32% in 24 h.

The agitation speed of dissolution apparatus for release study must be adjusted to 100 rpm [46, 47]. However, changing the stirring rate does not have any significant impact on release profile [48]. The findings of this study are also similar. The release of drug from patches subjected to agitation speed of 50 rpm, 75 rpm, and 100 rpm as specified in Figure 4(d) was 76.64 ± 1.24%, 76.77 ± 0.73%, and 79.73 ± 4.37%, respectively.

### 3.8. In Vitro Skin Permeation Study

**Figure 5:**
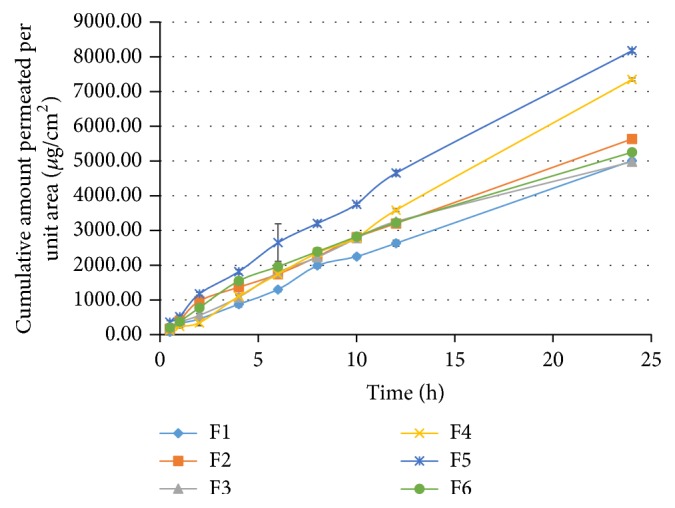
In vitro skin permeation profile of formulations F1–F6 from dexibuprofen reservoir patches.

The thickness of whole rat skin, 2.09 mm is approximately similar to human skin, that is, 2.97 mm [49]. The lipid content and water uptake of rat and human skin are also comparable. Rat skin contains 44.5% while human skin contains 45.1% lipid content. The water uptake of rat and human skin is 7.08 mg/mL and 8.32 mg/mL, respectively [50]. It is also proposed that the permeation pathway followed by stratum corneum of both human and rats is similar [51]. Thus, rat skin can be used as a surrogate for permeation studies. The permeation of dexibuprofen from reservoir patches F1–F6 across rat skin is presented in Figure 5. The cumulative amount of dexibuprofen permeated per unit area from F5 was found to be 8174.45 ± 54.26 *µ*g/cm^2^. In a study conducted by Prabu et al., 305 *µ*g dexibuprofen was permeated from optimized matrix patch of 1.5 cm^2^ in 24 h [9]. Similarly in another study, the flux of dexibuprofen from optimized matrix formulation was calculated and was found to be 206 *µ*g/cm^2^h [52]. However, formulation 5 exhibited highest permeation with flux 331.17 *µ*g/cm^2^h, which is higher than the previous transdermal formulations of dexibuprofen. The permeability coefficient of this formulation was 2.51*E* − 03 cm/h, lag time 1.33 h, and diffusion coefficient 3.74*E* − 05 cm^2^/h. These parameters for all formulations were computed and are presented in Table 5. The value of *F*_*D*_ for formulations was 0.953, 0.974, 0.956, 0.780, 0.861, and 0.941, respectively. The value of *F*_*D*_ closer to 1 indicates that the control resides majorly by device and contribution of skin in rate control is less [26].

**Figure 6:**
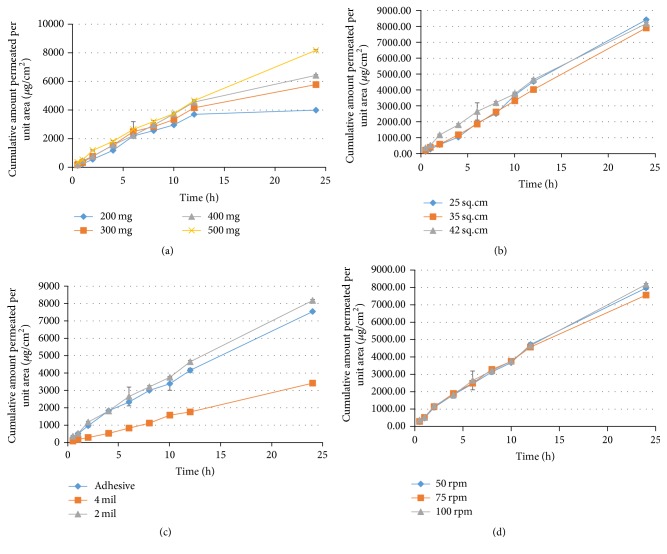
Effect of variable components on permeation of dexibuprofen from reservoir patch. (a) Effect of drug loading on permeation. (b) Effect of surface area on permeation. (c) Effect of transdermal components on permeation. (d) Effect of agitation speed on permeation.

The results as described in Figure 6(a) suggest that increase in drug loading leads to increase in rate of permeation. Parallel results were found by Pichayakorn et al. that higher nicotine loading resulted in higher permeation rate [29]. However, surface area does not have any significant impact on permeation. Approximately similar rate of permeation was found from patch of 25 cm^2^, 35 cm^2^, and 42 cm^2^, that is, 361.68 *µ*g/cm^2^h, 330.78 *µ*g/cm^2^h, and 331.17 *µ*g/cm^2^h, respectively, indicating that variation in surface area only affects the drug release.

Kim et al. concluded that use of Duro-Tak 85-2510 increases the permeation more as compared to other adhesives [53] due to its better adhesion property [54].  Figure 6(c) represents the permeation profile and it is found that the permeation from membrane coated with adhesive was 7535.41 ± 61.74 *µ*g/cm^2^ while without adhesive it was 8174.45 ± 54.26 *µ*g/cm^2^. The thicker membrane exhibited maximum permeation of 1119.83 ± 85.19 *µ*g/cm^2^.

Impact of agitation speed on permeation rate as mentioned in Figure 6(d) was also studied and it was observed that the rate of permeation for agitation speed of 50 rpm, 75 rpm, and 100 rpm was comparable. The flux at 50 rpm, 75 rpm, and 100 rpm was 327.29 *µ*g/cm^2^h, 308.74 *µ*g/cm^2^h, and 331.17 *µ*g/cm^2^h, respectively. Thus, no significant impact of varying agitation speed on permeation was observed.

### 3.9. Formulation Optimization

For optimization of dexibuprofen reservoir patch, simplex lattice design was used. The concentration of oil (*X*_1_), surfactant mixture (*X*_2_), and water (*X*_3_) were chosen as independent variables. The release of drug at 24 h (*Q*_24_) and flux of dexibuprofen across rat skin were taken as responses (*Y*_1_) and (*Y*_2_), respectively. the following equation describes the simplex lattice model used:

(12) Y=b1X1+b2X2+b3X3+b12X1X2+b13X1X3+b23X2X3,

where *Y* represents the independent variable and for the factor *X*_*i*_ the estimated coefficient is *b*_*i*_. The average result of changing single factor at a time from low to high value is presented by *X*_1_, *X*_2_, and *X*_3_ and the interactions *X*_1_*X*_2_, *X*_1_*X*_3_, and *X*_2_*X*_3_ represent the impact of changing two factors simultaneously.

On the basis of pseudoternary diagram, the ranges of components for construction of microemulsion were selected. *Q*_24_ and flux were measured as mentioned in Table 6. The results were computed through Design-Expert version 7 (Stat-Ease, Inc. Minneapolis) and are shown in

(13) YQ24=46.89X1+43.75X2+48.58X3+135.24X1X2+127.98X1X3−8.02X2X3YJ=213.69X1+210.55X2+227.5X3+390.4X1X2+442.26X1X3−39.54X2X3.

Equation (13) can be used to calculate predicted values of other formulation in the design space. The formulation shown in Table 2 was chosen to test the agreement between observed and predicted value as presented in Table 6. The predicted values of *Q*_24_ and flux for simplex lattice design were closer to those of experiment.

**Figure 7:**
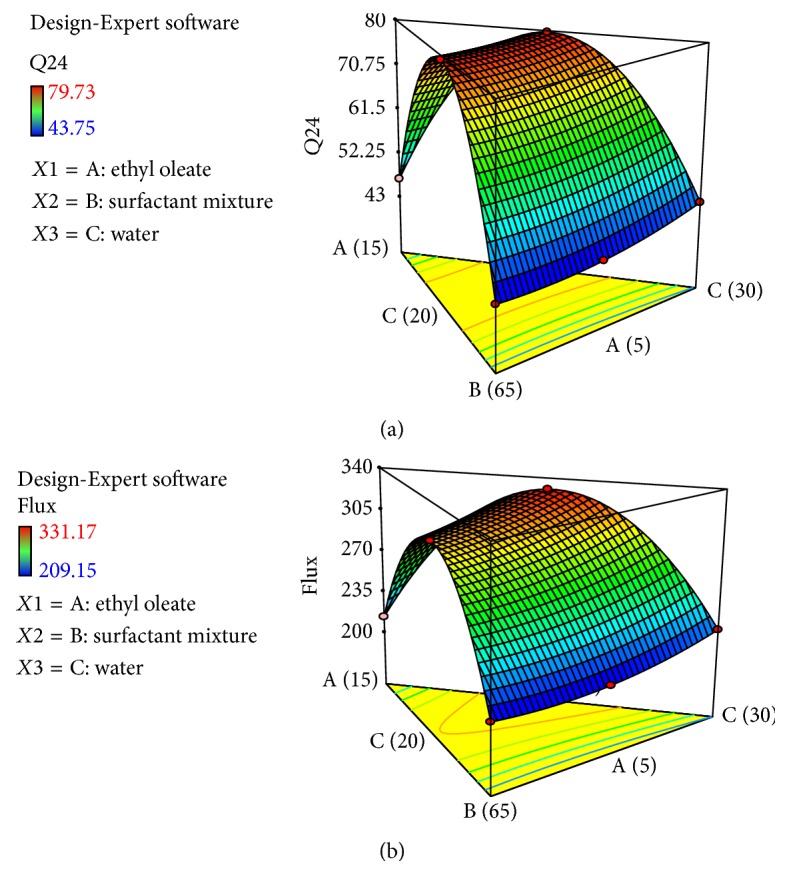
Response surface curve for optimization of dexibuprofen reservoir patches. (a) *Q*_24_. (b) Flux.

According to the results when the midvalue of oil and water was taken, the maximum release and permeation of dexibuprofen across rat skin were observed. The response surface curve of release of drug from patch and its flux was constructed and shown in Figure 7. Formulation F5 was selected as an optimized formulation on the basis of highest release and permeation rates.

### 3.10. Skin Sensitivity Reaction

**Figure 8:**
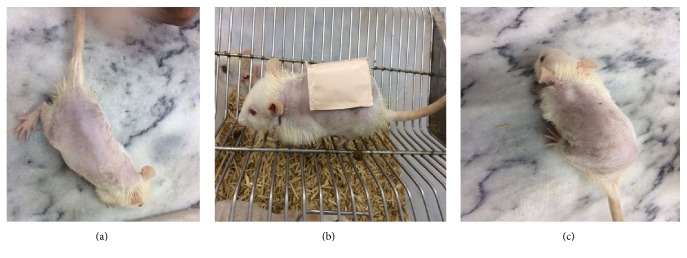
Skin sensitivity study of optimized dexibuprofen reservoir patch (*n* = 3). (a) Before application of patch. (b) Optimized patch applied. (c) After removal of patch.

Transdermal delivery systems have potential of causing irritation and allergic reactions [55]. These skin reactions must be studied and reported to determine the cause and to prevent the allergic reactions [56]. The pressure sensitive adhesive used for adhering the patch may lead to skin reactions. Therefore, investigating the skin sensitivity test by applying patch for recommended time period is essential [57].  Figure 8 represents the results of skin sensitivity reactions. No allergic reaction or irritation was observed after 24 hours of patch application. This indicates that the formulated patch is safe for use. Similarly, no visible irritation, erythema, or edema was observed when dexibuprofen matrix patches were applied on rabbit skin [9, 52].

### 3.11. In Vivo Anti-Inflammatory Activity

**Figure 9:**
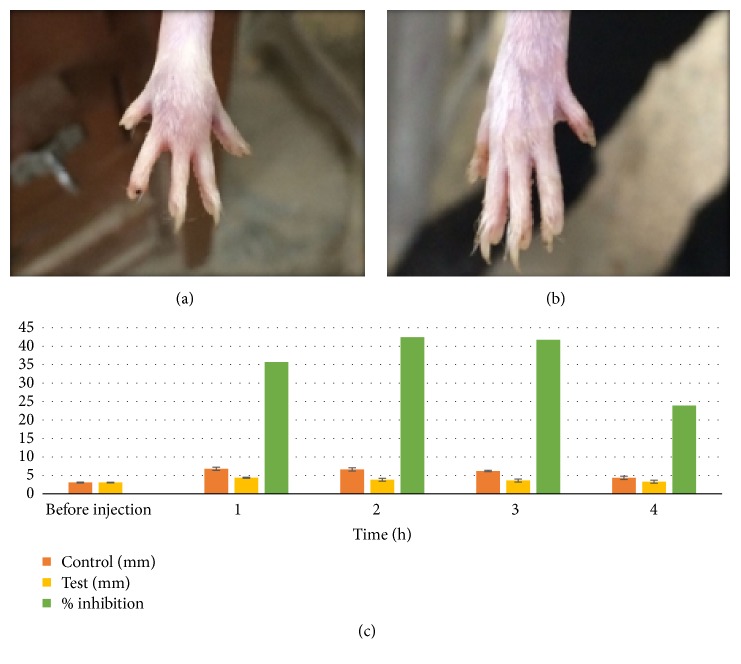
Anti-inflammatory activity of optimized dexibuprofen reservoir patch. (a) Hind paw of control rat. (b) Hind paw of test rat. (c) Graphical presentation of swelling of hind paw of control and test rat and % inhibition.

The commonly employed method for evaluating and screening anti-inflammatory activity is assessing the ability to inhibit edema produced by injecting phlogistic agent in hind paw of rat [58]. Carrageenan induces inflammation that is acute, nonimmune, and reproducible, which is characterized by increase in size of hind paw [59].  Figure 9 represents the control and test rat hind paw indicating the swelled paw in control rat while the test rat has no swelling. The percent inhibition of edema is also represented graphically indicating a significant reduction in hind paw swelling when dexibuprofen patch was applied. Similarly, it was concluded by Jin et al. that dexibuprofen emulsion gel has greater effectivity against carrageenan hind paw edema as compared to commercial hydrogel and ibuprofen emulsion gel [60].

### 3.12. Stability Studies

Dexibuprofen patch of 42 cm^2^ consisting 500 mg dexibuprofen (Formulation 5) was selected for stability studies at 3 different temperatures over a period of 3 months. The stability studies showed that the dexibuprofen content in patch after 3 months was 94.6 ± 2.54%, 88.77 ± 1.8%, and 86.9 ± 0.55% when kept at 4°C, room temperature, and 45°C, respectively. It was observed that the dexibuprofen content decreases after a long storage period at high temperature. Drug from the reservoir constantly migrates towards the periphery and as a consequence the drug content decreases [24].

The release and permeation of dexibuprofen from the patches stored for 3 months were studied and it was concluded that it also decreases with time especially when temperature is raised. The drug released after 3 months was found to be 65.18 ± 0.68%, 61.92 ± 0.61%, and 60.15 ± 0.375 at 4°C, room temperature, and 45°C, respectively. Higher temperatures have more impact on reduction of release rate. The decline in permeation rate is attributed to the reduction in release [29]. The absorption of lipophilic solvents by polymers of membrane leads to loss of permeation enhancers and also contributes to reduction of flux across human skin [61]. The flux of the patches kept at room temperature also declined from 0th to 3rd month and was found to be 333.17 *µ*g/cm^2^h, 325.60 *µ*g/cm^2^h, 315.37 *µ*g/cm^2^h, and 308.08 *µ*g/cm^2^h, respectively.

Environmental factors like oxygen, moisture content, and light have negative impact on stability of drug and permeation enhancers of transdermal patches; as a result the shelf-life is shorter [61]. Shelf-life for the dexibuprofen patches at different temperatures was also calculated and was found to be 6.14 months at 4°C, 3.12 months at room temperature, and 2.18 months at 45°C. Thus, storing the patch in refrigerator in order to prolong its shelf-life is suggested.

## 4. Conclusion

The reservoir-type transdermal patch exhibiting controlled zero-order rate of release with suitable permeation rate was prepared. The findings of in vitro studies suggest effective delivery of dexibuprofen across skin. This was also supported by the required in vivo anti-inflammatory activity. The developed patch with optimal quality attributes and no skin irritation or allergy observed when applied on skin for required time period can be used as a suitable alternative for administration of dexibuprofen and avoiding the adverse effects related to oral route of administration. This will be helpful in achieving steady-state plasma levels with a constant anti-inflammatory activity. It will also improve patient compliance.

## Figures and Tables

**Table 1:** Solubility of dexibuprofen in various vehicles.

Vehicle	Solubility (g/mL)
Ethyl oleate	0.182 ± 0.011
Surfactants	
Tween 40	0.165 ± 0.019
Tween 60	0.126 ± 0.023
Tween 80	0.306 ± 0.006
Triethanolamine	0.109 ± 0.013
Cosurfactants	
Propylene glycol	0.209 ± 0.026
Polyvinyl alcohol	0.133 ± 0.019
PEG 400	0.123 ± 0.012
Ethanol	0.105 ± 0.029
Oily mixture	0.536 ± 0.013

**Table 2:** The levels, composition, and responses of dexibuprofen microemulsion formulations computed by simplex lattice design.

Formulation	*X*_1_ (%)	*X*_2_ (%)	*X*_3_ (%)	pH	Conductance (*µ*S/cm)	Viscosity (mPa·S)	Refractive index	Size (nm)	PI	Drug content (%)
F1	5	60	25	4.66 ± 0.04	29 ± 0.17	360 ± 15.27	1.44 ± 0.001	138–175 ± 30	0.56	99.4 ± 2.48
F2	5	55	30	4.74 ± 0.09	32.7 ± 0.81	280 ± 20.8	1.44 ± 0.001	119.8 ± 13	0.4	100.8 ± 1.96
F3	5	65	20	4.94 ± 0.04	32.32 ± 0.92	250 ± 20.8	1.44 ± 0.001	221.6 ± 30	0.577	100.53 ± 0.83
F4	10	60	20	5.48 ± 0.26	12.94 ± 0.04	240 ± 15.27	1.45 ± 0.001	163–240 ± 30	0.36	97.9 ± 1.38
F5	10	55	25	5.46 ± 0.02	12.71 ± 0.02	110 ± 15.27	1.45 ± 0.001	160–186 ± 30	0.56	100.2 ± 0.35
F6	15	55	20	5.21 ± 0.04	20.13 ± 0.11	280 ± 20	1.45 ± 0.002	188–205 ± 40	0.35	99 ± 3.8

**Table 3:** Droplet size as a function of time for F5 microemulsion measured by DLS at 25°C.

Time (h)	Droplet size (nm)	Polydispersity index
0	55–133 ± 15	0.2
1	66–295 ± 25	0.2
2	248–282 ± 36	0.4
3	107–336 ± 25	0.2
4	251–287 ± 40	0.38
5	254–269 ± 39	0.4
6	259–291 ± 46	0.59

**Table 4:** Model fitting of the dexibuprofen reservoir patches (F1–F6) release profile.

Mathematical models	F1	F2	F3	F4	F5	F6
Zero order						
*R*^2^	0.9776	0.9797	0.9486	**0.9952**	**0.9985**	0.9690
*k*_0_ (h^−1^)	1.810	1.869	1.781	3.292	3.228	1.938

First order						
*R*^2^	0.9883	0.9621	0.9627	0.9467	0.9544	0.9873
*k*_1_ (h^−1^)	0.025	0.030	0.028	0.046	0.048	0.029

Higuchi model						
*R*^2^	0.8876	0.9473	0.9390	0.8011	0.8264	0.9136
*k*_*H*_ (h^−1/2^)	7.33	8.636	8.032	11.841	12.131	8.262

Korsmeyer-Peppas						
*R*^2^	0.9917	0.9958	0.9899	0.9951	0.9968	0.9926
*n*	0.8	0.677	0.680	0.993	0.944	0.743
*K*_kp_ (h^−*n*^)	3.476	5.579	5.512	3.385	3.940	4.526

Weibull						
*R*^2^	0.9914	0.9974	0.9950	0.9948	0.9978	0.9957
*T*_*d*_ (h)	44.413	38.54	48.698	18.080	18.066	39.57
*A*	31.56	29.004	19.572	244.148	410.054	25.744
*B*	0.911	0.916	0.766	1.810	1.935	0.883

Makoid Banakar						
*R*^2^	**0.9918**	**0.9976**	**0.9952**	0.9951	0.9982	**0.9962**
*n*	0.837	0.599	0.869	1.033	0.777	0.936
*k*_MB_	3.314	6.123	4.096	3.196	4.984	3.522
*K*	0.003	−0.007	0.016	0.003	−0.013	0.016

**Table 5:** Permeation parameters of formulations F1–F6 of dexibuprofen reservoir patches.

Formulations	Lag time (*t*_lag_)	Permeability coefficient (*P*)	Diffusion coefficient (*D*)	*F*_*D*_	*F*_*s*_	Best fit equation for permeation plot	Regression coefficient
	h	cm/h	cm^2^/h				
F1	0.42	1.18*E* − 03	1.18*E* − 04	0.953	0.047	*Q* = 209.15*t* + 87.9	0.9954
F2	1.56	1.58*E* − 03	3.19*E* − 05	0.974	0.026	*Q* = 227.52*t* + 1.56	0.9894
F3	1.59	1.52*E* − 03	3.12*E* − 05	0.956	0.044	*Q* = 210.55*t* + 334.99	0.9669
F4	0.46	1.90*E* − 03	1.08*E* − 04	0.780	0.220	*Q* = 309.72*t* − 141.12	0.9983
F5	1.33	2.51*E* − 03	3.74*E* − 05	0.861	0.139	*Q* = 331.17*t* + 439.73	0.9939
F6	2.15	1.34*E* − 03	2.31*E* − 05	0.941	0.059	*Q* = 213.69*t* + 458.84	0.9710

**Table 6:** Comparison of experimental results (mean ± SD; *n* = 3) and predicted values.

Formulations	*Q*_24_ (%)		*J* (*µ*g/cm^2^h)	
	Experimental	Predicted	Experimental	Predicted
F1	44.16 ± 0.46	44.16	209.14 ± 0.22	209.15
F2	48.58 ± 0.59	48.58	227.41 ± 4.42	227.52
F3	43.75 ± 4.5	43.75	210.55 ± 4.38	210.55
F4	79.13 ± 3.08	79.13	309.71 ± 1.41	309.72
F5	79.73 ± 4.37	79.73	331.17 ± 1.70	331.17
F6	46.89 ± 3.4	46.89	213.69 ± 1.52	213.69
